# A Project to Create a Novel Teaching Session on Gambling Harm, in Collaboration With Gamcare, for 4th Year Medical Students From Kings College London University (KCL) in Response to Changes in the Medical Licencing Assessment

**DOI:** 10.1192/bjo.2025.10301

**Published:** 2025-06-20

**Authors:** Rachel Rice, Hanna Mansi

**Affiliations:** KMPT, Kent, United Kingdom

## Abstract

**Aims:** Medical professionals are highly likely to come into contact with individuals who experience gambling harm during their careers. Around 5.5% of women and 11.9% of men globally experience some degree of gambling harm, and they are 15 times more likely to die by suicide than the general population. For every person that gambles around six others are affected. Our aim was to create a memorable teaching session involving people with lived experience of gambling harm.

**Methods:** The two-hour online teaching session was created in collaboration with Gamcare, a charity which supports individuals who are affected by gambling harm. Two individuals shared their experience of gambling harm; the first experienced significant gambling harm and attempted to end their life. The second was an affected other and highlighted their experience as a partner. The session was then followed by gambling training by one of Gamcare’s training leads.

**Results:** We had exceptionally positive feedback from the students with a highlight being the individuals who shared their lived experiences. Many students commented that the topic was one that they hadn’t had exposure to before this session. Students said they were able to learn the best ways to approach the conversation around gambling addiction and gained a better understanding of the signs of gambling harm.

**Conclusion:** Gambling harm is experienced by our patients regardless of our chosen speciality. This session delivered by providing students with a foundation of understanding which will enable them to open conversations and support individuals affected by gambling harm within their future practice.

